# *Histoplasma capsulatum* and *Pneumocystis* spp. co-infection in wild bats from Argentina, French Guyana, and Mexico

**DOI:** 10.1186/1471-2180-14-23

**Published:** 2014-02-05

**Authors:** Antonio E González-González, Cécile M Aliouat-Denis, José A Ramírez-Bárcenas, Christine Demanche, Muriel Pottier, Laura E Carreto-Binaghi, Haroon Akbar, Sandra Derouiche, Magalie Chabé, El Moukhtar Aliouat, Eduardo Dei-Cas, Maria Lucia Taylor

**Affiliations:** 1 Department of Microbiology and Parasitology, School of Medicine, National Autonomous University of Mexico, Mexico City 04510, Mexico; 2 Biology and Diversity of Emerging Eukaryotic Pathogens (BDEEP, EA4547), INSERM U1019, CNRS UMR8204, Institute Pasteur of Lille, Lille F-59019, France; 3 CHU Lille, Biology and Pathology Center, Parasitology-Mycology, Lille F-59000, France

**Keywords:** *Histoplasma*, *Pneumocystis*, Co-infection, Bats, PCR

## Abstract

**Background:**

*Histoplasma capsulatum* and *Pneumocystis* organisms cause host infections primarily affecting the lung tissue. *H. capsulatum* is endemic in the United States of America and Latin American countries. In special environments, *H. capsulatum* is commonly associated with bat and bird droppings. *Pneumocystis*-host specificity has been primarily studied in laboratory animals, and its ability to be harboured by wild animals remains as an important issue for understanding the spread of this pathogen in nature. Bats infected with *H. capsulatum* or *Pneumocystis* spp. have been found, with this mammal serving as a probable reservoir and disperser; however, the co-infection of bats with both of these microorganisms has never been explored. To evaluate the impact of *H. capsulatum* and *Pneumocystis* spp. infections in this flying mammal, 21 bat lungs from Argentina (AR), 13 from French Guyana (FG), and 88 from Mexico (MX) were screened using nested-PCR of the fragments, employing the *Hcp100* locus for *H. capsulatum* and the *mtLSUrRNA* and *mtSSUrRNA* loci for *Pneumocystis* organisms.

**Results:**

Of the 122 bats studied, 98 revealed *H. capsulatum* infections in which 55 of these bats exhibited this infection alone. In addition, 51 bats revealed *Pneumocystis* spp. infection of which eight bats exhibited a *Pneumocystis* infection alone. A total of 43 bats (eight from AR, one from FG, and 34 from MX) were found co-infected with both fungi, representing a co-infection rate of 35.2% (95% CI = 26.8-43.6%).

**Conclusion:**

The data highlights the *H. capsulatum* and *Pneumocystis* spp.co-infection in bat population’s suggesting interplay with this wild host.

## Background

Histoplasmosis due to *Histoplasma capsulatum* and pneumonia caused by *Pneumocystis* spp. are fungal diseases that can generate serious and even life-threatening pneumonia in immunosuppressed hosts. *H. capsulatum* is a fungal pathogen that affects a wide range of mammal species, including the human. Autochthonous clinical cases have been reported between the latitudes 54° 05′ North (Alberta, Canada) and 38° South (Neuquén, Argentina) [1,2]. The disease associated with this fungus is relevant in the geographical areas where histoplasmosis is endemic or epidemic, such as the Missouri, Ohio, and Mississippi river valleys, in the United States of America (USA), and some Latin American countries with a high frequency of outbreaks [3,4]. In Mexico, histoplasmosis is widely distributed and case reports are rather variable [4]. Infection is caused by the inhalation of fungal saprobe mycelial-phase propagules (infective form) that develop in special environments and are mainly found in bat guano accumulated in confined spaces such as caves and abandoned mines and buildings. The potential role of bats in spreading *H. capsulatum* in nature remains unclear. The high risk of natural bat infection with this fungus in Mexican caves has been well-documented [5-8]. According to their genetic diversities, *H. capsulatum* isolates from different geographical origins have been grouped into eight clades; seven of which are considered phylogenetic species. Among these, highlight the LAm A clade that harbours significant genetic variability [9].

The genus *Pneumocystis* contains highly diversified fungal pathogens that are harboured by a wide range of mammal hosts [10-16]. *Pneumocystis* organisms, which are transmitted via host-to-host airborne route, have a marked host-species-related diversity that is associated with close host specificity. The high divergence among *Pneumocystis* species most likely resulted from a prolonged process of co-evolution with each mammal host, mostly associated with co-speciation, as suggested by Demanche et al. [12] and Hugot et al. [13]. Although most phenotypic and genotypic data supporting *Pneumocystis* stenoxenism derives from laboratory animal models or captive animals, reports about *Pneumocystis* prevalence and circulation in wild fauna are scarce [12-16].

Unpublished preliminary data by our team revealed *H. capsulatum* and *Pneumocystis* co-infection in two randomly captured bats, identifying these mammals as probable reservoirs and dispersers of both parasites in nature (Dei-Cas E and Taylor ML, comm. pers.). The study of co-infection systems, where the host (i.e. a wild host) usually harbours two or multiple parasites, requires an in-depth investigation to determine a comprehensive understanding of this multi-infectious process in regards to its dynamics and consequences.

*H. capsulatum* and *Pneumocystis* share a number of features that justify their concomitant study, including: a low pathogenicity in healthy hosts and severe disease in immunocompromised hosts; an induced immune response; a respiratory portal of entry; and the ability to disseminate from the lungs to other organs. Therefore, it is possible that co-infection between both parasites is highly common in nature.

The aim of the present research was to detect the frequency of the *H. capsulatum* and *Pneumocystis* organisms’ infection and co-infection in the lung samples of a number of wild bat species from three countries from Latin America. For this purpose, we used a highly sensitive PCR with specific molecular markers for each pathogen that have been used successfully in clinical patients.

## Methods

### Bat samples

A total of 122 bats from different species and families were randomly captured as reported by Taylor et al. [7]: 21 came from Argentina; 13 came from French Guyana; and 88 came from Mexico. In all cases national rules regulating bat species protection, capture, and processing have adhered to strict ethical recommendations and to the guidelines published by Gannon, Sikes and the Animal Care and Use Committee of the American Society of Mammalogists [17].

The bats were euthanized by cervical dislocation and processed according to recommendations and approval of the Faculty of Medicine Ethics Committee, in accordance with the Animal Care and Use Committee of the UNAM and the Mexican Official Guide (NOM 062-ZOO-1999). The lungs from each bat captured in Mexico were separated and immediately frozen at −20°C. Animals captured in Argentina and French Guyana were also euthanized by cervical dislocation and processed *in situ* and their lungs were separated and preserved in 70% ethanol until DNA extraction.

### DNA samples

DNA was extracted from the bat lungs using a DNeasy Blood & Tissue Kit (Qiagen, Valencia, CA, USA). After extraction, the DNA samples were frozen at −20°C. The DNA samples were screened for *H. capsulatum* infection using nested-PCR for a fragment of the gene encoding a 100-kDa protein (*Hcp100*) [18], a molecular marker considered to be highly specific for this pathogen. Molecular screening for *Pneumocystis* spp. infection was conducted in parallel, using nested-PCR for fragments of the rRNA mitochondrial large [19,20] and small [21] subunit loci, *mtLSUrRNA* and *mtSSUrRNA*, respectively.

### Nested PCR assay of the *Hcp100* locus for the detection of *H. capsulatum*

The assay was performed as described by Bialek et al. [18] with minor modifications by Taylor et al. [22] that did not change the specificity and sensitivity of the *Hcp100* marker. Two sets of primers, described by Bialek et al. [18], were used: the outer primer set included HcI (5′-GCG-TTC-CGA-GCC-TTC-CAC-CTC-AAC-3′) and HcII (5′-ATG-TCC-CAT-CGG-GCG-CCG-TGT-AGT-3′); the inner primers were HcIII (5′-GAG-ATC-TAG-TCG-CGG-CCA-GGT-TCA-3′) and HcIV (5′-AGG-AGA-GAA-CTG-TAT-CGG-TGG-CTT-G-3′) and delimit a 210 base pair (bp) fragment unique to *H. capsulatum*. The primers were supplied by Operon Technologies Inc. (Alameda, CA, USA).

The first and second PCR reactions of the *Hcp100* locus were standardised elsewhere [6]. For the first round of amplification, the thermocycling conditions were as follows: one cycle at 94°C for 5 min; 35 cycles at 94°C for 30 s, 50°C for 30 s and 72°C for 1 min; and a final cycle at 72°C for 5 min. Thermocycling conditions for the second PCR (nested reaction) were: one cycle at 94°C for 5 min; 30 cycles at 94°C for 30 s, 65°C for 30 s and 72°C for 1 min; and a final extension cycle at 72°C for 5 min. The DNA (20 ng) of the EH-53 *H. capsulatum* strain from a Mexican clinical case was used as a positive amplification control, and Milli-Q water was processed as a negative control.

### Nested PCR assays of the *mtLSUrRNA* and *mtSSUrRNA* loci for the detection of *Pneumocystis* spp

The assays were based on amplifying fragments of the mitochondrial large (*mtLSU*) and small (*mtSSU*) subunits of the rRNA gene.

Nested PCR at the *mtLSUrRNA* locus employed the outer primer set published by Wakefield et al. [19], pAZ102-H (5′-GTG-TAC-GTT-GCA-AAG-TAG-TC-3′) and pAZ102-E (5′-GAT-GGC-TGT-TTC-CAA-GCC-CA-3′). The inner primers pAZ102-X (5′-GTG-AAA-TAC-AAA-TCG-GAC-TAG-G-3′) and pAZ102-Y (5′-TCA-CTT-AAT-ATT-AAT-TGG-GGA-GC-3′) and delimit a 267 bp fragment for *Pneumocystis*[20]. The first and nested PCR reactions of the *mtLSUrRNA* locus were standardised as described elsewhere [14,19,20]. For the first round of amplification, the thermocycling conditions were as follows: 30 cycles at 94°C for 30 s, 50°C for 1 min, and 65°C for 1 min. The nested reaction was performed with 10% of the first-round amplification product and the thermocycling conditions were: 30 cycles at 94°C for 30 s, 55°C for 1 min, and 65°C for 1 min.

Nested PCR at the *mtSSUrRNA* locus was performed with the outer primers, pAZ112-10 F (5′-GGG-AAT-TCT-AGA-CGG-TCA-CAG-AGA-TCA-G-3′) and pAZ112-10R (5′-GGG-AAT-TCG-AAC-GAT-TAC-TAG-CAA-CCC-3′). The inner primers pAZ112-13RI (5′-GGG-AAT-TCG-AAG-CAT-GTT-GTT-TAA-TTC-G-3′) and pAZ112-14RI (5′-GGG-AAT-TCT-TCA-AAG-AAT-CGA-GTT-TCA-G-3′) and delimit a 300 bp fragment for *Pneumocystis* species, as reported by Tsolaki et al. [21]. PCR for the *mtSSUrRNA* locus was previously described by Tsolaki et al. [21] and Akbar et al. [14]. For the first round of amplification, the thermocycling conditions were as follows: 40 cycles at 94°C for 30 s, 55°C for 1 min, and 65°C for 1 min. The nested round was performed with 10% of the first-round amplification product and the thermocycling conditions were: 10 cycles at 94°C for 30 s, 52°C for 1 min, and 65°C for 1 min, followed by 30 cycles at 94°C for 30 s, 63°C for 1 min, and 65°C for 1 min.

Primers for the *mtLSUrRNA* and *mtSSUrRNA* loci were supplied by Operon Technologies. The DNA (20 ng) of rabbit *Pneumocystis* (*Pneumocystis oryctolagi*) was processed as a positive amplification control, and Milli-Q water was used as a negative control for both *Pneumocystis* molecular markers.

### Amplified products

Amplicons from each PCR assay were electrophoresed through 1.5% agarose in 0.5X Tris-borate-EDTA buffer. Electrophoresis was conducted at 120 V for 50 min. The 100 bp DNA Ladder (Gibco Laboratories, Grand Island, NY, USA) was used as a molecular size marker. The bands were visualised using a UV transilluminator after ethidium bromide staining (0.5 μg/mL). The amplicons were purified using the QIAquick*®* PCR and the QIAEX II kits (Qiagen) for the *H. capsulatum* and *Pneumocystis* organisms, respectively. Afterwards, the amplicons were sent to the Molecular Biology Laboratory, Institute of Cellular Physiology (UNAM, Mexico) for sequencing in an ABI-automated DNA sequencer (Applied Biosystems Inc., Foster City, CA, USA). Sequencing reactions were performed for forward and reverse DNA strands, and a consensus sequence for each amplified bat lung sample product was generated. The sequences were edited and aligned using the MEGA software, version 5 (http://www.megasoftware.net).

Most of the *Hcp100* sequences of *H. capsulatum* were previously reported in González-González et al. [6], and the other sequences were deposited in a database [GenBank: from JX091346 to JX091370 accession numbers]. All sequences generated by both molecular markers for *Pneumocystis* spp. were reported by Derouiche et al. [16] and Akbar et al. [14].

The sequences of the specific markers for each pathogen (i.e., *Hcp100* for *H. capsulatum* and *mtLSUrRNA* or *mtSSUrRNA* for *Pneumocystis* spp.) that were obtained in the same animal were the main inclusion criterion for considering bat co-infection.

### Statistics

The infection and co-infection rates for each pathogen were estimated by considering all of the bats studied from the three countries and from each country separately (Argentina, French Guyana, and Mexico), in relation to those bats with *H. capsulatum* and *Pneumocystis* spp. infections as identified by sequencing their respective molecular markers. The corresponding 95% confidence interval (CI) was calculated using a normal distribution.

## Results

Data from nine bat species studied belonging to five different families, highlighting their particular behaviours, such as migration, nourishment, distribution in the American continent and colony size, are referred to in Table 1, according to Ceballos and Oliva [23]. These behaviours varied considerably among the bat species studied (Table 1). The different species captured, their numbers, and their geographical origins are registered in Table 2. Although most of the bat species studied were non-migratory, the number of migratory bats from three processed species was greater than that of the non-migratory species (Tables 1 and 2). It is noteworthy that among the 122 bats studied, 84 (68.80%) belonged to the migratory species *Tadarida brasiliensis*, from which 63 individuals were captured in Mexico and 21 in Argentina (Table 2).

**Table 1:** Families, species, and behaviours of the bats studied

**(Family) Species**	**Migration**	**Nourishment**	**Distribution**	**Colony size**
(Phyllostomidae)				
*Artibeus hirsutus*	Non-migratory	Frugivorous	From south of Sonora to south of Guerrero, Mexico	Not defined
*Carollia perspicillata*	Non-migratory	Frugivorous	From Tamaulipas to Oaxaca, Mexico, and to south of Bolivia, Brazil and Paraguay	Small groups from 10 to 100 individuals
*Glossophaga soricina*	Non-migratory	Nectarivorous, polinivorous, frugivorous and insectivorous	From Mexico to South America	From a few to 2,000 individuals
(Natalidae)				
*Natalus stramineus*	Non-migratory	Insectivorous	From north of Mexico to Brazil	Approximately 10,000 individuals
(Mormoopidae)				
*Pteronotus davyi*	Non-migratory	Insectivorous	From north of Mexico to Brazil	From hundreds to thousands of individuals
*Pteronotus parnellii*	Non-migratory	Insectivorous	From north of Mexico to Brazil	From hundreds to thousands of individuals
*Pteronotus parnellii*	Non-migratory	Insectivorous	From north of Mexico to the north of Argentina and Paraguay	Thousands of individuals
*Mormoops megalophylla*	Migratory	Insectivorous	From south USA to Venezuela and Peru	From a few to thousands of individuals
(Molossidae)				
*Tadarida brasiliensis*	Migratory	Insectivorous	From central USA to Chile and Argentina	Generally, thousands to millions of individuals
(Vespertilionidae)				
*Myotis californicus*	Occasionally migratory	Insectivorous	From western Canada and USA to Guatemala	Small groups or gregarious

Data from the bat species were reported by Ceballos and Oliva [23].

**Table 2:** **Species, numbers, and geographical origins of the bats infected with *H. capsulatum *** **or *Pneumocystis *** **spp.**

**Species**				**Geographical origins/localities**			**Number of bats infected with *H. capsulatum*/*Pneumocystis***			
	**Argentina**		**French Guyana**	**Mexico**						**(Total samples per species)**
	**TUC**	**CBA**	**Kourou**	**CS**	**MN**	**GR**	**HG**	**MS**	**NL**	
***H. capsulatum*/*Pneumocystis *** **(total samples)**										
*A. hirsutus*								5/3 (5)		5/3 (5)
*C. perspicillata*			1/0 (1)							1/0 (1)
*G. soricina*			3/6 (12)			4/3 (4)				7/9 (16)
*N. stramineus*						5/1 (8)				5/1 (8)
*P. davyi*						1/0 (1)				1/0 (1)
*P. parnellii*						2/0 (2)	0/1 (1)			2/1 (3)
*M. megalophylla*						2/0 (2)			1/0 (1)	3/0 (3)
*T. brasiliensis*	16/8 (16)	3/ND (5)		8/2 (8)	7/2 (8)		13/5 (20)		26/19 (27)	73/36 (84)
*M. californicus*									1/1 (1)	1/1 (1)
Number of bats infected with *H. capsulatum*/*Pneumocystis* (Total samples per locality)	16/8 (16)	3/ND (5)	4/6 (13)	8/2 (8)	7/2 (8)	14/4 (17)	13/6 (21)	5/3 (5)	28/20 (29)	98/51 (122)

Abbreviations: TUC = Tucumán; CBA = Córdoba; CS = Chiapas; MN = Michoacán; GR = Guerrero; HG = Hidalgo; MS = Morelos; NL = Nuevo León.

ND = Not determined.

### Detection of *H. capsulatum* infection in the bat lung samples

**Figure 1:**
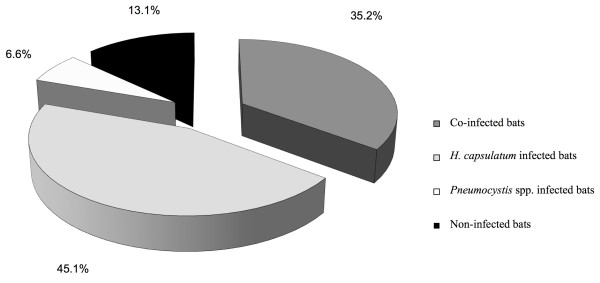
**Percentages of *H. capsulatum *and *Pneumocystis *infection and their respective co-infection in bats randomly sampled in Argentina, French Guyana, and Mexico.** Each percentage value was calculated based on the 122 bats captured. Bat infection was screened with specific molecular markers for each pathogen, as described in the Methods section.

Of the 122 bat lungs that were molecularly screened for *H. capsulatum* infection, 98 bats generated sequences for the *Hcp100* marker, of which 55 bats were found to be infected with this pathogen alone, corresponding to 45.1% (95% CI = 36.4-53.6%) of the 122 bats from the three geographical regions studied (Figure 1).

Table 2 displays the number of infected bats with *H. capsulatum* in relation to the total number of each bat species sampled at different localities from the monitored Latin American countries.

### Detection of *Pneumocystis* spp. infection in the bat lung samples

Of the 122 lungs that were molecularly screened for *Pneumocystis* spp., 51 bats generated sequences for one or both of the *Pneumocystis* molecular markers assayed. From these sequences, seven matched the *mtLSUrRNA* locus and another seven matched the *mtSSUrRNA* locus, while 37 sequences were generated at both loci. *Pneumocystis* spp. infection alone was found only in eight bats*,* corresponding to 6.6% (95% CI = 2.25-10.85%) of the total bats studied (Figure 1).

Table 2 displays the number of infected bats with *Pneumocystis* spp*.* in relation to the total number of each bat species sampled at different localities from the monitored Latin American countries.

### *H. capsulatum* and *Pneumocystis* spp. co-infection in the bat lung samples

Of the lung samples from the 122 bats captured in Argentina, French Guyana, and Mexico that were molecularly screened for *H. capsulatum* and *Pneumocystis* infections, 43 samples revealed the specific sequences of each pathogen, corresponding to 35.2% (95% CI = 26.8-43.6%) of the samples being co-infected with both pathogens in bats from the three geographical regions studied (Figure 1).

Table 3 displays the number of co-infected bats with both pathogens in relation to the total number of each bat species sampled at different localities from the monitored Latin American countries.

**Table 3:** **Species, numbers, and geographical origins of the bats co-infected with *H. capsulatum *** **and *Pneumocystis *** **spp.**

**Species**	**Geographical origins/localities**									
	**Argentina (n = 21)**		**French Guyana (n = 13)**	**Mexico (n = 88)**						**Number of co-infected bats**
	**TUC**	**CBA**	**Kourou**	**CS**	**MN**	**GR**	**HG**	**MS**	**NL**	**(Total samples per species)**
**Co-infection (total samples)**										
*A. hirsutus*								3 (5)		3 (5)
*C. perspicillata*			0 (1)							0 (1)
*G. soricina*			1 (12)			3 (4)				4 (16)
*N. stramineus*						1 (8)				1 (8)
*P. davyi*						0 (1)				0 (1)
*P. parnellii*						0 (2)	0 (1)			0 (3)
*M. megalophylla*						0 (2)			0 (1)	0 (3)
*T. brasiliensis*	8 (16)	0 (5)		2 (8)	2 (8)		3 (20)		19 (27)	34 (84)
*M. californicus*									1 (1)	1 (1)
Number of co-infected bats (Total samples per locality)	8 (16)	0 (5)	1 (13)	2 (8)	2 (8)	4 (17)	3 (21)	3 (5)	20 (29)	43 (122)

Abbreviations: TUC = Tucumán; CBA = Córdoba; CS = Chiapas; MN = Michoacán; GR = Guerrero; HG = Hidalgo; MS = Morelos; NL = Nuevo León.

Finally, of the total number of bat lungs sampled, 106 (86.8%, 95% CI = 80.92-92.68%) were found to be infected with one or both pathogens, whereas 16 (13.1%, 95% CI = 7.22-18.98%) did not show evidence of infection with any of the pathogens studied (Figure 1).

## Discussion

The co-infection relationship between the host and different parasite species could occur in natural conditions, although it has been scarcely studied due to its complexity and poor understanding [24]. The presence of more than one parasite species in a single host can lead to positive or negative interactions. In the positive interaction, the parasite could favour the entry and survival of another parasite, whereas in the negative interaction the establishment of a parasite prevents the entry of other parasites and abolishes their survival [24]. It is well accepted in medical research that the infection concept implies the presence of the pathogen in the infected host’s tissues, which does not necessarily indicate a disease status that is supported by characteristic signs and symptoms. Although bats, in general, have a high infection rate with *H. capsulatum* in their shelters, they most likely do not develop a severe course of the disease [7], and the impact of this infection on the survival of their population is unknown. With regard to *Pneumocystis* bat infection, this wild host could present a latent infection without evidence of any disease signs and symptoms [12,14]. Consequently, bats could be potential carriers of both parasites in the environment.

*H. capsulatum* and *Pneumocystis* spp. cause a host infection through the respiratory airway, mainly affecting the pulmonary tissue. After infecting the lungs, each parasite develops on distinct host environments and exploits different host resources. The *H. capsulatum* parasitic yeast-phase is an intracellular pathogen of the lung phagocytic cells. In contrast, *Pneumocystis* organisms are extracellular pathogens that frequently attach to type I pneumocytes [10].

*Histoplasma*-*Pneumocystis* co-infection has been reported in immunosuppressed human patients [25], whereas reports of co-infection in wild mammals have not been published. This fact should be re-examined because both parasites are able to share the same wild hosts in a particular manner, likely associated with the host immune status related to stress, sickness, and nutrient starvation.

PCR assays that utilize specific molecular markers are very sensitive tools for detecting a low fungal burden in clinical samples from asymptomatic patients. Currently, *H. capsulatum* and *Pneumocystis* spp. infections are detected by different PCR methods, either in human clinical cases or in experimental models [14,26-29]. The present study is the first report for detecting a natural co-infection in wild bats from three distant geographical Latin American regions, using specific PCR assay for each parasite.

The numbers of wild bats infected with *H. capsulatum* or *Pneumocystis* organisms varied, with the number of *H. capsulatum* infected bats surpassing the number of *Pneumocystis* infected bats (Figure 1). No association was found between a bat species’ susceptibility and nourishment and the rate of infection with both pathogens. However, it is possible that some bat behaviours promote different infection rates for either *H. capsulatum* or *Pneumocystis* spp. According to published findings, the rates of each pathogen infection could be associated with the bat colony size and their movements, in the case of *H. capsulatum*[7], or with behavioural factors such as bats crowding and migration in the case of *Pneumocystis* spp. [14]. The biggest colonies, mainly of *T. brasiliensis*, had the highest rate of infection with *H. capsulatum*, most likely due to bat colony movements within enclosed spaces, especially in shelters where short ceiling-to-floor distances prevails, which facilitate the development of a great number of airborne infective propagules on the abundant guano accumulated underneath bat colonies [7]. Hence, each of these factors allows the co-infection state with both pathogens.

Based on the following evidence, it is likely that either *H. capsulatum* or *Pneumocystis* displayed an interaction with different bat species since million of years ago (Ma): 1.- Bat fossils (*Tadarida* sp.) were reported approximately 3.6 – 1.8 Ma in the Late Pliocene [30]; 2.- the *H. capsulatum* complex most likely started its radiation at 13–3 Ma in the Miocene [9]; and 3.- the *Pneumocystis* species have had interaction with mammal hosts for more than 100 Ma [10-13,31]. Under this assumption, the co-infection of both pathogens most likely generated a co-evolution process between each pathogen and the wild host.

Data pertaining to *Histoplasma-Pneumocystis* co-infection reveal a rate of 35.2%; this finding could be useful for understanding the persistence of both infections in susceptible hosts. The absence of *Histoplasma* or *Pneumocystis* infections in 13.1% of the bats studied could suggest that most of the analysed bat populations were exposed to a high risk of infection with these pathogens in their shelters. Co-infection interactions could cause ecological and immunological implications for the host. For the ecological implications, space and alimentary competitions are involved. For the immunological implications, the host immune response against *H. capsulatum* at the pulmonary level involves cells and molecules that could also participate in the host immune response against *Pneumocystis,* or vice versa.

## Conclusion

The impact of the present findings highlights the *H. capsulatum* and *Pneumocystis* spp. co-infection in bat population’s suggesting interplay with this wild host. In addition, this co-infection state could also interfere with the outcome of the disease associated with each pathogen.

## Competing interests

The authors declare that they have no conflicts of interest.

## Authors’ contributions

MLT and EDC contributed equally to the design of this study. AEGG coordinated and performed the molecular assays for *H. capsulatum* detection. MLT and AEGG contributed equally to draft the manuscript. JARB and LECB processed the bat samples from Argentina and Mexico and collaborated in the molecular assays for *H. capsulatum*. EDC, ELMA, CMAD, CD, and MC coordinated the molecular assays of *Pneumocystis* and revised the manuscript draft. MP, HA, and SD performed molecular assays for Pneumocystis detection. All authors have read and approved the manuscript.
